# Preventing incisional hernia: closing the midline laparotomy

**DOI:** 10.1007/s10151-018-1833-y

**Published:** 2018-08-09

**Authors:** M. M. J. van Rooijen, J. F. Lange

**Affiliations:** Department of Surgery, Erasmus University Medical Centre Rotterdam, Rotterdam, The Netherlands

## Abridged abstract

### Background

There is ongoing debate as to which suturing techniques and suture materials are best for achieving definitive abdominal wound closure while minimising the risk of short- and long-term complications.

### Objectives

The objectives of this review were to identify the best available suture techniques and suture materials for closure of the fascia following laparotomy incisions, by assessing the following comparisons: absorbable versus non-absorbable sutures; mass versus layered closure; continuous versus interrupted closure techniques; monofilament versus multifilament sutures; and slow absorbable versus fast absorbable sutures.

### Search strategy

On 8 February 2017 we searched CENTRAL, MEDLINE, Embase, two trials registries, and Science Citation Index. We included randomized controlled trials (RCTs) that compared suture materials or closure techniques, or both, for fascial closure of laparotomy incisions.

### Main results

Fifty-five RCTs with a total of 19,174 participants met the inclusion criteria and were included in the meta-analysis. Included studies were heterogeneous in the type of sutures used, methods of closure and patient population. Many of the included studies reported multiple comparisons.

For our primary outcome, the proportion of participants who developed incisional hernia at 1 year or more of follow-up, we did not find evidence that suture absorption (absorbable versus non-absorbable sutures, RR 1.07, 95% CI 0.86–1.32, moderate-quality evidence; or slow versus fast absorbable sutures, RR 0.81, 95% CI 0.63–1.06, moderate-quality evidence), closure method (mass versus layered, RR 1.92, 95% CI 0.58–6.35, very low-quality evidence) or closure technique (continuous versus interrupted, RR 1.01, 95% CI 0.76–1.35, moderate-quality evidence) resulted in a difference in the risk of incisional hernia. We did, however, find evidence to suggest that monofilament sutures reduced the risk of incisional hernia when compared with multifilament sutures (RR 0.76, 95% CI 0.59–0.98, *I*^2^ = 30%, moderate-quality evidence).

For our secondary outcomes, we found that none of the interventions reduced the risk of wound infection, whether based on suture absorption (absorbable versus non-absorbable sutures, RR 0.99, 95% CI 0.84–1.17, moderate-quality evidence; or slow versus fast absorbable sutures, RR 1.16, 95% CI 0.85–1.57, moderate-quality evidence), closure method (mass versus layered, RR 0.93, 95% CI 0.67–1.30, low-quality evidence) or closure technique (continuous versus interrupted, RR 1.13, 95% CI 0.96–1.34, moderate-quality evidence).

Similarly, none of the interventions reduced the risk of wound dehiscence whether based on suture absorption (absorbable versus non-absorbable sutures, RR 0.78, 95% CI 0.55–1.10, moderate-quality evidence; or slow versus fast absorbable sutures, RR 1.55, 95% CI 0.92–2.61, moderate-quality evidence), closure method (mass versus layered, RR 0.69, 95% CI 0.31–1.52, moderate-quality evidence) or closure technique (continuous versus interrupted, RR 1.21, 95% CI 0.90–1.64, moderate-quality evidence).

Absorbable sutures, compared with non-absorbable sutures (RR 0.49, 95% CI 0.26–0.94, low-quality evidence) reduced the risk of sinus or fistula tract formation. None of the other comparisons showed a difference (slow versus fast absorbable sutures, RR 0.88, 95% CI 0.05–16.05, very low-quality evidence; mass versus layered, RR 0.49, 95% CI 0.15–1.62, low-quality evidence; continuous versus interrupted, RR 1.51, 95% CI 0.64–3.61, very low-quality evidence).

## Commentary

Understanding how to prevent incisional hernia after midline laparotomy is paramount for surgeons, gynecologists, and urologists. When closure after midline laparotomy fails, several complications can occur. Most importantly, the patient will develop an incisional hernia, accompanied by a reduction in quality of life and potential reoperation with additional costs. To prevent patients from experiencing complications, available materials and techniques should be considered and evaluated systematically, as was done in the Cochrane review by Patel et al. [1].

A number of important factors, however, are not reported in the included studies of this current review. First, the primary outcome of incisional hernia was only measured at 1 year. As shown by a number of authors, nearly half of incisional hernias occur later than in the first year after surgery [2–4]. Therefore, the review provides insufficient evidence about the long-term effects of the researched materials and techniques. Second, the studies included in the review had considerable heterogeneity. In the primary analysis, all information is taken together: no distinction was made between emergency and elective procedures, and although a subgroup analysis was done for midline incisions, this was not done for paramedian or other incisions. The follow-up was done either clinically, by ultrasound, or both, while small incisional hernias can easily be missed clinically. Additionally, the review shows no adjustment for patient risk factors, such as age, body mass index, or chronic obstructive pulmonary disease. This heterogeneity leads to diluted effects, making it difficult to draw definite conclusions about single patient groups.

Last and most importantly, the review is entitled “closure methods”, but it overlooks some important factors involved in closing the midline, which definitely influence the development of incisional hernia.

First of all, regarding suturing techniques, in several large randomized trials of more than 500 patients it has been shown that small bite size sutures—as first reported by Israelsson [5]—can reduce the development of incisional hernias [6, 7]. Small bites have shown a higher bursting pressure than large bites: if the tension on the wound is distributed over a large number of stitches, the tension on each stitch will be low [8].

Second, the use of mesh reinforcement has not been addressed in this review. In high-risk patients, for example the obese or patients with an abdominal aortic aneurysm (AAA), there is ample clinical evidence now that prophylactic mesh augmentation after midline laparotomy can decrease incisional hernia development, regardless of whether it is placed sublay or onlay [9]. Both patient groups suffer from compromised collagen synthesis; patients with AAA are at risk of incisional hernia due to a possible underlying connective tissue disorder, and obesity is associated with wound healing complications due to decreased vascularization of the adipose tissue and an increase of proinflammatory tissue factors. However, two questions still remain about the use of mesh reinforcement. One is whether prophylactic mesh augmentation should become standard practice in all patients, and the second is what kind of mesh material to use. Regarding the first question, the risk of complications with mesh (e.g. seroma, infection) should be weighed against the benefit of preventing incisional hernia in every single patient. Regarding what kind of material should be used, mostly the choice is between (slowly) resorbable or permanent mesh. The resorption rate of synthetic or biological meshes is critical: too rapid resorption does not support sufficient healing and might not effectively prevent the development of incisional hernia. Non-resorbable synthetic mesh, however, is more prone to infection. The latest development is represented by slowly absorbable synthetic mesh, hypothesized to “remodel” the abdominal wall. However, for now, too little is known about this type of mesh, and more research into the effectiveness and safety of this material is needed.

Since infection is a risk factor for incisional hernia, the prevention of infection in midline wounds is important, especially in high-risk patients. The prophylactic use of negative pressure wound therapy might have an important role here, yet too little is known about the effectiveness of this method as a preventative measure.

All the above-mentioned factors can influence the development of incisional hernia. Research into these topics should be continued. However, investigating new techniques and materials is difficult. Clinical studies are expensive and per se unsuitable for investigating new methods, whereas preclinical experiments with animals render limited evidence, since the abdominal wall anatomy is considerably different from that of humans. A fairly recently developed artificial abdominal wall simulator is the AbdoMAN, representing a physical model that mimics the internal and external forces on the abdominal wall quite realistically. On this model, several suturing techniques and materials can be tested in a high fidelity simuleted setting. This can offer advantages for midline repair research, as no patients or animals are needed in the exploration of the biomechanics of new theories, techniques, and materials [10].

So what can be said about augmentation materials and suturing techniques in midline laparotomies? As the Dutch surgeon Hans Jeekel said, “Closure time is no coffee time.” Closing a laparotomy should be taken as seriously as all prior operative steps, and it should be performed by a dedicated surgeon, who closes only the linea alba, in order to prevent necrosis of muscle tissue due to sutures. From the evidence presented in the review [1], patients could profit from the use of monofilament sutures. However, patients could perhaps benefit even more from the small bite size technique and from prophylactic mesh augmentation. Depending on the patient risk factors and the contamination of the wound, the most suitable mesh material needs to be identified. Time will tell whether slowly resorbable synthetic mesh lives up to its early promise. The role of prophylactic negative pressure wound therapy seems promising but more data are needed. Although the serious complication of incisional hernia can never be fully prevented due to factors such as surgeon experience and patient risk factors, more research into old and newly developed materials and techniques is still necessary.
